# Dental Orthopœdia

**Published:** 1855-01

**Authors:** A. F. Talma, F. H. Rehwinkel

**Affiliations:** Dentist to the King of Belgium.


### ARTICLE II.

*Dental Orthopœdia.* By DR. A. F. TALMA, Dentist to the King of Belgium. Translated from "Der Zahnarzt," by F. H. REHWINKEL.†

INFERIOR MAXILLA.—In some instances the incisors of the lower jaw protrude so far as to overlap those of the upper. The cutting edge, and anterior surface of the latter, are in such cases soon worn off, and a groove is formed, which in a short time will extend to the nerve cavity, thereby exposing the dental pulp, causing the organ to ache, and eventually, making its re-

† The number containing the first part of this article having failed to reach us, we are unable to publish it at this time. We may do so hereafter should it come to hand.—EDS.

moval necessary. In other cases, the cutting edge of the incisors of the superior maxilla come in contact with the posterior surface of the lower gums, producing great irritation, and this, usually results in ulceration—when this takes place, the teeth must necessarily become loose in their sockets.

The above named deformity may depend either upon the anomalous direction, anteriorly, of the incisors of the lower maxilla, whilst those of the upper are in a regular and correct position; or the teeth of the superior maxilla may incline so much inwardly as to be quite behind the circle of the lower jaw. It is a matter of great importance to distingush between these two irregularities; and this can only be done by a very careful examination, which must take place in early youth; for in later years, when the teeth are fully grown, the constant pressure to which they have been subjected will have changed their original positions, and make the first cause, or starting point, exceedingly difficult to ascertain. If it is found, that the deformity consists in the protrusion of the lower teeth, which is not unfrequently the case, even if those instances are excepted in which pressure is produced upon the teeth by hard tumors under the tongue, and which force them beyond the upper dental arch; it is advisable, in the first place, to direct our operations principally to the deviating organs. For the attainment of his purpose, the dentist must, after the removal of the cause, or complication of causes, which may be the occasion of the irregularity, commence his operations upon the lower dental arch, by the same means which I have heretofore recommended, for the correction of deformities in the teeth of the superior maxilla; namely, the direct pressure, the plates, the bars, and ligatures. When the latter are adopted, they must be attended to carefully, and their action modified and regulated to the indications of the case. When the lower teeth are in a normal position, and the upper show the inclination to fall behind the former, circumstances must guide the choice of means, used to correct the deformity.

I cannot repeat too often, that a deviation should be recognized in the beginning, and the proper measures taken for its correction. Should a case occur, in which the irregularly inclining teeth are yet too short to come in contact with those of the inferior maxilla, it may be hoped, that a methodical pressure of the thumb and fingers will suffice to force them into a correct position; more especially as the constant and unremitting pressure of the tongue will materially assist.

When these simple means have been neglected, and the upper teeth already strike the lower, we may succeed in remedying the evil, by filing their respective edges in such a manner as to cause them to meet obliquely, and permit the upper teeth to close over the lower. This method, however, can be considered applicable only in two cases, on account of the loss of substance, and the danger of serious consequences ensuing, if the inclination inwardly should amount to more than three millimeters. With many persons, this difficulty may be avoided, if the antagonists which stand in an anomalous position are prevented coming in contact with each other.

Duval, whose experience has often guided me, recommends a gold or platina plate, with which the lower circle is cased, and which is fashioned in the shape of an inverted groove or gutter, and fastened on either side to the molar teeth.

The crossbars, highly recommended by Delabarre, are in some cases more efficient than the plan of Duval. The fixture is applied and secured in the same manner, as the one already described.

Either one of these methods will prevent the entire closing of the teeth, and the pressure of the thumb and fingers, with the assistance of the tongue, are, as we have before observed, oftentimes all that is necessary, to compel the fractious little fellows to resume their proper situation. The foregoing contrivances have the advantage over some others, of doing no injury to the teeth by loosening them, and the dentist merely assists nature in accomplishing the desired end. There are cases, in which the faulty position of the teeth has been of such long standing, that an immense amount of pressure would be required, to correct the deformity; rendering it impossible to accomplish the object in the above described manner, as the necessary fixtures would be too cumbersome, to be worn with any degree of comfort.

For these cases, which have been considered almost incurable, Catalan, an ingenious and successful practitioner, once my instructor, and whose friendship, I have now the honor of possessing, has invented an apparatus, known under the name of the "inclined plane." This in a kind of cap, formed over the lower teeth and fastened to them, and in which they are of course enclosed. On this cap are soldered projecting pieces of gold or platina, which incline inwardly in an oblique position, that the posterior surface of the incisors of superior maxilla may touch the *upper part* of the inclined plane.

The mode of operation of these planes will be at once understood, from the above description, and it must be clear to every one, that the lower teeth, provided with these weapons, can sooner or later, compel the upper to assume their normal positions. As soon as the offending teeth have so far regained their regular places, as to strike slightly over those of the lower maxilla, these artificial means can be dispensed with; for the teeth themselves will, without further assistance, complete the cure. It is indispensably necessary frequently to examine the apparatus, in order to ascertain if the action is too powerful, and if such is the case, to adjust and modify it; for this would not only be extremely inconvenient to the patient, but even dangerous. To obviate this difficulty, it is advisable to apply the instrument so that at first the pressure will be slight, and gradually increase until the object is attained. Many have strongly objected to Catalan's invention, but I am inclined to believe that all difficulty has been occasioned by their not understanding its proper application. It is quite incorrect that the teeth, which are by these means forced into regular positions, become loose, thereby causing fears, lest they should be altogether lost. The galvanic action, to which some persons object, exists only in their own imaginations. The sensibility of the teeth, occasioned by the planes, is in some cases so great, as to render the little patient unable to masticate his food. This, however, lasts but a few days, and is never present if the apparatus has been properly applied. It is also a great mistake to suppose that it is instrumental in causing or promoting caries of the teeth, or ulceration of the gums. When these take place, we may feel sure, that the operator has not been sufficiently careful in his application of the instrument, or that he has been neglectful during its use.

It is above all things necessary, to be certain that the dispositions of the parts, will permit the employment of the inclined plane. Furthermore, it is indispensable that it should be an accurate fit. If, for instance, it should be inaccurately or carelessly fitted, and permitted to rock upon the teeth, as a matter of course, they would soon become loosened, thus causing it to become a positive evil.

Delabarre has substituted for the cap, or inverted groove, two metal plates, which fit anteriorly and posteriorly, with great exactness, to the teeth; having them united at each end with a clasp, which encloses the last molars. From the front plate projects inwardly and in an oblique direction, a piece of gold or platina, making precisely the inclined plane. This alteration, seems to me more of a complication than an improvement.

The advantage which he claims for this method over that of Catalan is, that there is much less liability of its retaining portions of food, and the secretions of the mouth; thereby preventing the softening of the enamel, or caries of the teeth.

These evils are easily obviated by attention to the ordinary means of cleanliness. The two clasps appertaining to the so-called improvement, are very liable to fatigue the molars around which they are fastened, and by their want of firmness will allow the plates to rock upon the front teeth and thus loosen them. In short, the inclined plane of Catalan is, in my estimation, in all those cases demanding artificial means of this kind, the most simple and easy of application, of which dentistry can boast.

It is from long experience, that I commend this method, and not as may be supposed, from prejudice, or to the exclusion of others which may be equally good. If, for example, as I have heretofore remarked, the deformity lies in a great lateral inclination, and unusual protrusion of the front incisors of the lower maxilla, it will often be proper to operate directly on those teeth, and particularly when the teeth of the superior maxilla are in a normal position. Although this plan has been much lauded by some very eminent dentists, who have recommended its adoption in the generality of cases, nevertheless it is my opinion, that, like the last named, it is only applicable in extraordinary and peculiar instances.

There is another plan from which I have obtained great benefit in my own practice. A narrow strip of strong plate is fitted to the posterior surface of the lower circle, and secured to the molar teeth. This plate is perforated with pin holes opposite those teeth which incline too much outwardly. The rim must stand a little off from these teeth, which should have strong silken cords around them, the ends being passed through the holes in the plate. Thus in tying, we can bring to bear any amount of pressure desirable. In my long practice, I have met with many cases, which were so complicated as to render it necessary to use, both the "direct pull," and "inclined plane;" the former, to prepare the way, as it were, and the latter to complete the cure. Among the numerous instances which I might cite to uphold my views, I will only mention the following one.

The lower teeth protruded so far beyond the already projecting upper incisors, that their position was almost horizontal, giving to the countenance an exceedingly disagreeable expression. Want of room rendered it entirely impossible to bring the lower teeth back to their normal situation.

I, therefore, removed a bicuspid on each side, and by means of ligatures and wedges of gum elastic, induced the cuspidati to occupy the places thus vacated, and so gained the necessary space. After this preparation had been made, I proceeded to use the above mentioned means, and in a short time, not a trace of the deformity, which had rendered my little patient and her family almost despairing, was left. A short time previous, I had met with the same satisfactory success in two similar cases.

The dentist should never hesitate to sacrifice one or more of the bicuspids, when the regulation of protruding incisors cannot be accomplished without it. Of course, it must never be done, unless absolutely necessary. Even when the advantages expected from the removal of those teeth resolve themselves into nothing, the loss is not serious, for the molars naturally incline towards the centre, and will soon fill up the vacancy thus left. And there is in such cases even one advantage attained; the wisdom teeth make their appearance earlier, assume a better position, and are not as liable to decay. I must here remark, that the inclined planes, although their action is principally upon the upper teeth, are not without effect on the lower—as the same impulse which causes the former to come forward, also induces the latter to fall back. The object which the authors have in view by this method, will, in this way, partially at least, be accomplished in the most simple and rational manner, and against its too general application only do I object. As soon as a normal position of both arches is obtained, the cure is completed by a natural advancement of the upper teeth, and retreat of the lower, caused by reciprocal pressure. In my application of the inclined planes, I have made one observation, which in regard to theory is highly remarkable, and as far as practice is concerned, interesting.

When the apparatus has been worn for some time, say several months, the molars will grow more rapidly, in consequence of not striking their proper antagonists, and become proportionately longer than the other teeth. To prevent this difficulty, which would not only be a new deformity, but also be an obstacle to the proper mastication of the food, I would advise the removal of the apparatus before eating, (this would also be conducive to cleanliness,) and even for several hours during the day or night; thus making its action intermittent. If one or more frontal teeth of both jaws incline wrongly, the method in question, properly varied and adapted to each particular case, will always be applicable. The operator should before applying the planes, ascertain if there is sufficient room for the return of the teeth to their correct position, if not, the evil must be remedied, either by filing the neighboring teeth, or removing one or more of the bicuspids, as heretofore stated. The choice between these two means, must depend upon the relative situation of the parts. If but a small loss of substance will suffice, by use of the file, we can attain the object. If on the contrary, the teeth are crowded and the space completely filled up, extraction is indispensable.

In some instances, when the neighboring teeth of those which had deviated were at a considerable distance from each other, and the patient not more than twelve or fifteen years of age, I have succeeded in avoiding both operations, and have seen the teeth assume their correct places, pushing the interfering teeth out of their way, until the whole dental arch gradually became perfectly regular. Such is not often the result, but it proves the necessity of carefully studying the deformity before deciding upon the proper remedial agents. Ligatures, either of silk or wire, tied around the neck of the teeth, and from thence, running either in front or behind the neighboring teeth, and fastened to the molars, were for a long time exclusively used, for the regulation of the last mentioned deformities. Nothing better was known, but now, their universally acknowledged disadvantages, when compared with the late inventions, have induced most of the dental practitioners to discontinue their use. The crossbars and inclined plane, I have endeavored fully to describe, so that each dentist can be able to judge, in which particular cases, their several applications may be most expedient.

*Lateral Inclination.*—Some teeth while inclining either in or outwardly from the circle, also lean too much to the right or left, as the case may be. This irregularity is usually the result of premature extraction of some of the deciduous teeth; thus giving the deviating teeth unnecessary room at the time of their eruption. The most simple and effective manner of remedying this evil, is by means of ligatures fastened to the molars, the ends being carried, one within the circle, the other around it, to the deviating tooth, and secured to it in the best manner for accomplishing the desired end. If the tooth, in its inclination, is occupying space which should belong to another which has not yet appeared, when this begins to grow, it will materially assist in forcing the usurper to its own proper place. In such cases, the dentist has merely to assist nature's own efforts.

Another form of irregularity, is, when the approximal surfaces present a labial and lingual view. The best measures for removing this difficulty, are the variously arranged ligatures. Before making the attempt, we should satisfy ourselves of there being a sufficiency of room for turning the tooth. When this is found not to be the case, extracting one of the bicuspids will remove the difficulty. After complying with this primary indication, we proceed by winding a strong silken cord around the fractious organ, of which one end must come over, and the other under, or behind the adjoining tooth, and from thence be carried to the molars and there strongly fastened. The pressure should be constant and uniform, without being at any time too powerful, as this might produce irritation and other unpleasant consequences. Hence it is necessary, not only to be extremely careful in applying the ligatures, but also that they should be frequently examined, and righted, if occasion requires. In such cases, Delabarre recommends a capsule made of gold plate, and fitting accurately over the tooth. To this is attached a small tube, in which, by means of a screw, is secured one end of a bent lever—the other being carried round the anterior surface of the circle, and fastened to a bicuspid, or molar. This, it will at once be perceived, must exert great power upon the capsule, and consequently upon the enclosed tooth. It is a very ingenious contrivance, and can, in many cases, be advantageously substituted for the ligatures. Some daring practitioners, have expressed themselves in favor of the luxation of the tooth. This, however, is so frequently productive of evil to the organ itself, that it may be well considered wholly irrational. Notwithstanding the greatest care, it is often impossible to avoid fracturing the tooth in an attempt at luxation and turning. It is also evident that when a tooth is extracted or luxated, and again forced back into its socket, the adaptation of the parts is materially injured; the teeth no longer fit in the alveoli, sometimes causing extreme irritation. None the less true is it, that in consequence of the rotary motion, the nerves and vessels which communicate with the nerve cavity, and nourish the teeth, are necessarily turned, if not broken; if the latter, of course the organ becomes a foreign body, which sooner or later must be removed.

These weighty considerations, have, as a general thing, prevented my following this method; though in two instances I was persuaded to yield my better judgment to the entreaties of the parents of my young patients. I should not have done so, had not the chances of success been more than ordinarily favorable. Although the result far exceeded my anticipations, and was in every respect satisfactory, I do not hesitate to pronounce the operation extremely hazardous.

After correcting irregularities by rational means, the exercise of the proper functions of the teeth, will be sufficient to consolidate them, and complete the cure. Not so, after luxation. In this case it will be necessary to support the organ in its newly acquired position, for a considerable length of time, until it is again firmly imbedded in its socket. For each of my patients, I caused to be constructed, a gutter similar to that of the inclined plane, which they were required to wear at night, and as they fitted accurately over all the other teeth, the regularity of the latter was by these means preserved.

*General consideration of Ligatures, and other remedial Agents of Dental Orthopœdia.*—A few remarks on this subject, will not, I presume, be considered out of place.

To commence with ligatures, which are usually, either silken cords or light wire. I have already pointed out the disadvantages connected with their use; they are as follows: Their action is not confined to the organ for which they are used, but exerts an influence upon several: viz. those which are used for the support of the apparatus, those to which it is secured, and even those with which it merely comes in contact, must more or less suffer, in consequence of being brought to lean upon each other. Also, the pressure from so small a body is quite liable to injure the teeth, their substance being still of too soft and yielding a nature to resist the strong traction brought to bear upon them. Finally, of whatever material the ligatures may be composed, they are, to a greater or less extent, apt to slide down under the gums, thereby producing pain and inflammation, and partially lifting the teeth from their sockets, which in the generality of cases leads to their extraction.

To prevent this slipping of the cords, some have used a small hook, in this manner: one end is bent over the grinding surface of the tooth; the other, pointing upwards from the gum and the cord resting in the last bend. The complication of such apparatus, together with the difficulty of firmly securing it to the teeth, forms, in my estimation, sufficient reason for discarding it entirely, and considering it furthermore both useless and objectionable. I might say the same of the metal rings, which have been proposed for accomplishing the same object. They also, only serve to complicate the apparatus, and multiply the difficulties attending its employment.

For these hooks and rings, I have generally substituted a durable cord, which is so secured to the middle of the tooth that to slide down would be an impossibility. To this same knot I then unite the cord of traction. This has always proved successful, when the confirmation of the teeth would permit its application. Notwithstanding all the disadvantages which I have been enumerating, I do not mean to be understood that the use of ligatures in orthopœdic treatment should be entirely condemned—but the many facts which have come under my observation, during my long practice, induce me to advocate their use only in cases of extreme necessity, and when less severe measures have proved ineffectual.

For instance, in cases of lateral inclination, or rotation, or when the alveolar border is fully developed, and the inclined plane has been used without accomplishing the desired end, they may, as I have already shown, be brought into requisition. Especially if the object is merely to direct the inclination of the neighboring teeth, in order to make room for a deviating organ. When I speak in favor of ligatures, I mean of course only those of silk, or some vegetable substance, not those of a metallic nature, as they should never be used, excepting as a support for already adjusted teeth, and even then they cannot be employed without danger to *these*, as well as those to which they are fastened. The silken cords, used for this purpose, and with the necessary precautions, are quite harmless, as the fluids of the mouth keep them soft and swollen. The principal requisite in the use of ligatures, is their judicious application. The traction should be very moderate at first, and gradually increased. Especial care must be taken that they be not too painful. It is unnecessary, and fills the patient with dread and apprehension. In many cases, silken ligatures are advantageously superseded by wedges of gum-elastic properly introduced between the teeth.

It is now almost universally used, when it will at all answer the purpose, and possesses one very valuable property, viz. its expansion is gradual and uninterrupted, yet powerful enough to move the teeth. In this it is far superior to any thing of the kind as yet known.

The vulcanized gum-elastic is the most suitable for dental purposes, as it may be applied in almost any way, and lose none of its peculiarities. At first the layers should be extremely thin, and every two or three days exchanged for thicker ones, and continued until the object is attained. It must always be applied in such a manner as to adapt itself readily to the crown of the tooth, without coming too much in contact with the gums. In this, every thing depends upon the practical skill of the dentist. If the little patient is much annoyed, the cause can generally be traced either to too thick a wedge, or that it presses heavily upon the gum.

In all such cases it is essentially necessary to suspend operations until all symptoms of irritation have disappeared.

When several teeth are to be straightened, or forced into other places than those which they occupy, the dilating body must be so applied that it shall have a firm resting point on one of several teeth, (that they support each other,) whilst the one to be moved must be so situated that pressure of this kind can be brought to bear upon it. After the tooth has been regulated, the wedge can be taken out and applied in the same manner to the next one, and so on until the whole object has been accomplished.

By proceeding in this gradual and sure way, and adding to the action of the wedges, that of light ligatures, teeth may be moved to a considerable distance without producing any unpleasant consequences.

Compared with each other, the ligatures, the clasps, the elastic wedges, and even the inclined planes, have their disadvantages as well as the opposite. And it remains for the dentist to be guided by circumstances in his choice, as well as to use sound judgment for that which would be excellent in one case might prove naught in another.

*Crowding of the Teeth.*—Even if the situation of the teeth is perfectly normal, it not unfrequently happens that too much pressure, each upon the other, requires the interference of art. By some it has been considered a point of beauty to have a slight space between the teeth, discernible. Without expressing any opinion on this subject, I will merely consider the question from its hygienic point of view. According to this, the approximate surfaces of the teeth may slightly touch, but should never press upon each other. It must not be forgotten that however firm they seem, they are more or less liable to be pressed out of their correct position. When the whole dental apparatus is normally arranged, touching, slightly, not pressing, each tooth finds support from its neighbor, and the firmness of the whole circle is materially increased. On the other hand, when this symmetrical arrangement is disturbed, and a tooth is isolated in its position, or perhaps has been extracted, the firmness has been broken, and the other teeth in its neighborhood will become fatigued, and sometimes loosened in their sockets, and may finally, drop out. Too much pressure, however, as I have before mentioned, causes irritation at the point of approximation, and will result in caries, and final destruction of those teeth. Under these circumstances, it is of the utmost importance to consider carefully the real requirements of the teeth, before doing any thing to, or for them. It would be perfectly barbarous to separate the teeth merely to gratify a coquetish desire for beauty, when there was no indication of the operation being necessary, and in direct opposition too to the dignity of science. The separation of teeth by means of a file, is only justifiable when there are sure symptoms, or rather, evidences, of caries—(bluish spots on the enamel and between the teeth.) Where this is the case no time should be lost in performing the operation, as the evil increases daily. The longer the postponement, the greater the loss of substance, or if wholly neglected the caries would approach the nerve cavity, thus rendering filling necessary; even to give the tooth one chance of being preserved from destruction.

An unequaled length of the teeth is early remedied by filing those which are longer than the others, until they all correspond.

The objections against this mode of operation, have long since been dissolved into nothing; therefore, it is unnecessary to defend it. As a preventive of caries, as an orthopœdic, and finally as a preparatory remedy, it is of great service.

*Transposition of the Teeth—Supernumerary Teeth.*—Under this head, I comprise all those cases in which teeth are developed at a distance from their natural position, also such as are entirely extra. It is well worth while for the dental surgeon to pay strict attention to these cases, as they not unfrequently cause morbid affections in the mouth, as well as entirely disturbing the economy of the dental arches.

A man 45 years of age, who was destitute of the incisors and cuspidati of the lower jaw, had above the chin, on the base of the gum a fistulous wound, caused by an inflammatory swelling which had lasted some ten months. The abscess from which the fistula had developed itself, had thrown off two incisors. The still open wound, discharged a great quantity of matter of a tolerably good quality. The whole region of the lower jaw, and particularly inside of the mouth, was much swollen; which swelling, from time to time, became aggravated, accompanied by vague and deep seated pain. On probing the wound, I discovered two very hard and smooth substances, which I supposed to be teeth. The conclusion was confirmed on the preceding details being related to me. The teeth had in all probability deviated from their natural direction, and were still retained within the jaw.

The only remedy for the permanent relief of the patient was the removal of the teeth. To do this, as can well be imagined, was an exceedingly difficult operation. I was obliged to enlarge the fistula and dissect the gums from the alveolar process, thereby exposing a large portion of the maxilla. An opening was then made into the bone, and through this I succeeded in extracting four teeth. First a lateral incisor, then the two cuspidati, and finally another lateral incisor.

These teeth were almost horizontally imbedded in the jaw, and their position in other respects so irregular that the crown of the right cuspidatus corresponded with the root of the other. In the course of a few weeks, the wound entirely healed, and the cure was complete. It is evident that for such cases no general rules can be laid down, each dentist must use his own judgment, and contrive a method best suited to the disposition of the parts.

If transposed or supernumerary teeth have become permanently fixed, and obtained an equal development with the normal teeth, the skillful dentist will always be guided in his operations by the degree of obstruction produced upon the functions of the latter. He will also consider the dangers and difficulties attending their extraction, though in many cases this may take place without causing any serious disturbance.

I have seen instances in which a considerable part of the maxilla was exposed, and the ridge opened, without hindering, or prolonging, the healing of the parts. The opposite would most likely be the case, in unskillful operations.

Desirabode relates the case of a young man who had a wide opening made in the palate, which, in consequence of a fracture of the intermediate parts, communicated with the cavity of the nose; the whole, caused by an unskillful extraction of a supernumerary tooth. He was obliged to wear an obturator, the application of which was exceedingly difficult. This is merely one case, but it is sufficient to show, that "dental art" in the hands of an ignorant and unskillful man will suffer much. Dental orthopœdia must in its practical application be governed by general rules, which may be as follows: as much as possible to preserve and improve, and only in extreme cases to mutilate; proceeding always carefully, patiently and attentively; ever remembering the injunction of our oldest masters—*Principiis obsta.*

To be continued.

